# Regulation of tissue growth in plants – A mathematical modeling study on shade avoidance response in *Arabidopsis* hypocotyls

**DOI:** 10.3389/fpls.2024.1285655

**Published:** 2024-02-28

**Authors:** Patrick Favre, Evert van Schaik, Martine Schorderet, Florence Yerly, Didier Reinhardt

**Affiliations:** ^1^ Department of Biology, University of Fribourg, Fribourg, Switzerland; ^2^ Haute école d’ingénierie et d’architecture Fribourg, Haute Ecole Spécialisée de Suisse Occidentale (HES-SO), University of Applied Sciences and Arts of Western Switzerland, Fribourg, Switzerland

**Keywords:** *Arabidopsis thaliana*, shade avoidance syndrome, auxin, auxin transport, mathematical modeling, hypocotyl, morphogen, gradient

## Abstract

**Introduction:**

Plant growth is a plastic phenomenon controlled both by endogenous genetic programs and by environmental cues. The embryonic stem, the hypocotyl, is an ideal model system for the quantitative study of growth due to its relatively simple geometry and cellular organization, and to its essentially unidirectional growth pattern. The hypocotyl of Arabidopsis thaliana has been studied particularly well at the molecular-genetic level and at the cellular level, and it is the model of choice for analysis of the shade avoidance syndrome (SAS), a growth reaction that allows plants to compete with neighboring plants for light. During SAS, hypocotyl growth is controlled primarily by the growth hormone auxin, which stimulates cell expansion without the involvement of cell division.

**Methods:**

We assessed hypocotyl growth at cellular resolution in Arabidopsis mutants defective in auxin transport and biosynthesis and we designed a mathematical auxin transport model based on known polar and non-polar auxin transporters (ABCB1, ABCB19, and PINs) and on factors that control auxin homeostasis in the hypocotyl. In addition, we introduced into the model biophysical properties of the cell types based on precise cell wall measurements.

**Results and Discussion:**

Our model can generate the observed cellular growth patterns based on auxin distribution along the hypocotyl resulting from production in the cotyledons, transport along the hypocotyl, and general turnover of auxin. These principles, which resemble the features of mathematical models of animal morphogen gradients, allow to generate robust shallow auxin gradients as they are expected to exist in tissues that exhibit quantitative auxin-driven tissue growth, as opposed to the sharp auxin maxima generated by patterning mechanisms in plant development.

## Introduction

In many plants, the cotyledons are the first photosynthetic organs which need to be exposed to the sunlight as early as possible to ensure survival of the seedling. As an essential adaptation to varying environmental conditions during germination, the embryonic stem (the hypocotyl) shows distinct growth patterns in response to the direction, intensity, and quality of incoming light (Pierik and de Wit, 2014; de Wit et al., 2016). These external cues impinge on hormonal pathways, in particular on auxin, which control hypocotyl growth, thereby exposing the cotyledons to optimal light conditions (Pierik and de Wit, 2014; de Wit et al., 2016).

Auxin has two main biological functions in plant life: firstly, it is a growth hormone that can regulate tissue elongation in a quantitative way, e.g. in dicot hypocotyls or in grass coleoptiles (de Wit et al., 1970; Woodward and Bartel, 2005); secondly, auxin can act as a morphogen in developmental patterning and fate decisions (Leyser, 2005; Benkova et al., 2009; Vanneste and Friml, 2009). This latter function concerns various developmental states and plant organs, from embryogenesis to the formation and positioning of roots, leaves, flowers, and fruits (Benkova et al., 2003; Friml et al., 2003; Reinhardt et al., 2003; Blilou et al., 2005; Tanaka et al., 2006; Mironova et al., 2017; Shi and Vernoux, 2019). The action of auxin as a morphogen has attracted major attention of developmental biologists as well as theoreticians who modeled how auxin gradients are influenced by auxin biosynthesis, transport, and degradation (Barbier de Reuille et al., 2006; Jönsson et al., 2006; Smith et al., 2006; Newell et al., 2008; Stoma et al., 2008; Mirabet et al., 2012; Cieslak et al., 2015; Hartmann et al., 2019; Cieslak et al., 2021; Hartmann et al., 2021).

A central feature that characterizes the biology of auxin is its specific transport system that involves cellular import and export proteins, which determine the direction of auxin transport, thereby determining its distribution and accumulation in plant tissues (Geisler, 2021). Numerous studies have shown that this cellular transport system can generate dynamic auxin gradients with high spatial and temporal resolution (Benkova et al., 2003; Friml et al., 2003; Petersson et al., 2009; Galvan-Ampudia et al., 2020). Such gradients require active transport against an auxin gradient and could not be achieved by passive diffusion, as it is the case in classical models of morphogen function (Wartlick et al., 2009).

Mathematical models of auxin-dependent patterning usually involve local reinforcement (autocatalytic auxin accumulation) and lateral inhibition at a distance (auxin depletion in adjacent tissues) (Barbier de Reuille et al., 2006; Jönsson et al., 2006; Smith et al., 2006). Such models can recreate similar developmental patterns as models based on reaction-diffusion mechanisms, although the underlying mechanisms are fundamentally different (Smith and Bayer, 2009). Models based on auxin transport can recreate many of the patterns found in nature, for example in phyllotaxis, where auxin regulates organogenesis in space and time, thereby creating stunning spiral patterns defined by Fibonacci numbers (Smith et al., 2006; Reinhardt and Gola, 2022). Models of vascular patterning involve auxin flux as an additional factor to account for the tendency of auxin to become confined to narrow cell files that represent routes of high auxin flux, a phenomenon known as auxin canalization (Smith and Bayer, 2009; Biedron and Banasiak, 2018).

A central element of most patterning models is the active transport of auxin against concentration gradients, resulting in steep auxin gradients that mediate differential developmental decisions in the tissues. Such auxin gradients have been visualized by local accumulation of auxin reporters such as DR5 (Ulmasov et al., 1997). In root tips, for example, or at sites of leaf formation in the shoot apical meristem, local accumulation of DR5 coincides with the site of the root meristem, and of the leaf primordium initials, respectively, indicating that auxin accumulates at these sites (Sabatini et al., 1999; Benkova et al., 2003).

In contrast to its action as a morphogen, the action of auxin as a growth hormone does not involve major changes in cell identity, but it rather causes quantitative changes in cell volume, resulting in differential tissue growth. Auxin-driven cell expansion has been studied in various cell types and tissues including protoplasts (Steffens and Luthen, 2000; Dahlke et al., 2017), coleoptiles (Kutschera and Khanna, 2020), and hypocotyls (Fendrych et al., 2016; Kohnen et al., 2016; Reed et al., 2018). The nature of quantitative hypocotyl elongation suggests that, in contrast to the sharp auxin peaks involved in patterning phenomena, rather subtle changes in auxin levels and distribution can be expected to control growth patterns. As a special case of differential quantitative growth, unequal distribution of auxin across the diameter of the hypocotyl is thought to be the basis for phototropic growth towards unilateral light (Friml et al., 2002; Hohm et al., 2014).

One of the best-characterized examples of auxin-dependent growth phenomena, the shade avoidance syndrome (SAS), induces hypocotyl growth, allowing plants to grow out of the shade from neighboring plants (Pierik and de Wit, 2014; de Wit et al., 2016). Shade is perceived by phytochrome as a shift in light quality, resulting from the depletion of red light (R, 640-700 nm) that is absorbed by the shading plant, in contrast to far red light (FR, 700-760 nm) which is reflected, scattered or transmitted by shading leaves (de Wit et al., 2016). Hence, plants perceive shading as a decrease in the R:FR ratio. This allows growth to be controlled experimentally with high spatial and temporal precision. Therefore, the SAS has been the target of numerous genetic studies on hypocotyl growth in particular in *Arabidopsis* (Vandenbussche et al., 2005; Casal, 2013; de Wit et al., 2016).

The SAS of seedlings is based primarily on hypocotyl elongation, and involves only cell expansion but not cell division (Gendreau et al., 1997). The hypocotyl represents a relatively simple tubular structure that grows essentially unidirectionally (only in length), a growth phenomenon that is easier to quantify than more complex organs such as leaves. Cell length of hypocotyl epidermis is initially uniform, but hypocotyl growth results in a particular cellular growth pattern. Cells in the middle of the hypocotyl are longer than cells at its lower and upper end (Gendreau et al., 1997), suggesting that hypocotyl growth is uneven. The significance of this growth pattern, and the factors that govern growth at the cellular level are unknown. Also, it is not clear how plants may generate the shallow auxin distribution patterns along the hypocotyl that could explain the observed cellular growth patterns.

Here, we take a combined experimental and mathematical modeling approach to address how auxin biosynthesis, transport, and catabolism could generate gradients that correspond to the quantitative growth patterns observed in the *Arabidopsis* hypocotyl. Inspired by the finding that auxin homeostasis depends not only on auxin biosynthesis and transport, but also on auxin conversion and catabolism (Takase et al., 2004; Pencik et al., 2013; Zheng et al., 2016; Casanova-Sáez et al., 2021), we propose a model that invokes dynamic regulation of auxin biosynthesis and degradation in a context of constitutive basipetal and centrifugal polar auxin transport in the *Arabidopsis* hypocotyl. A central tenet of this model is that hypocotyl growth is limited by the outer epidermal cell wall layer, which is much thicker than the inner cell walls (Derbyshire et al., 2007), and which has previously been identified as the tissue that restricts, and therefore controls, elongation growth (Kutschera and Niklas, 2007; Savaldi-Goldstein et al., 2007). Our model shows that a mechanism that is controlled primarily by auxin production and degradation, in combination with constant polar fluxes, is sufficient to produce auxin gradients that correlate with the observed cellular growth patterns in *Arabidopsis* hypocotyls. While the molecular-genetic components of hormonal regulation of SAS have been elucidated in considerable detail (Martinez-Garcia and Rodriguez-Concepcion, 2023), a coherent quantitative description of the phenomenon is missing. Our mathematical model provides a global quantitative framework that links cellular auxin biology with growth patterns in SAS.

## Results

### Growth patterns in the *Arabidopsis* hypocotyl

**Figure 1:**
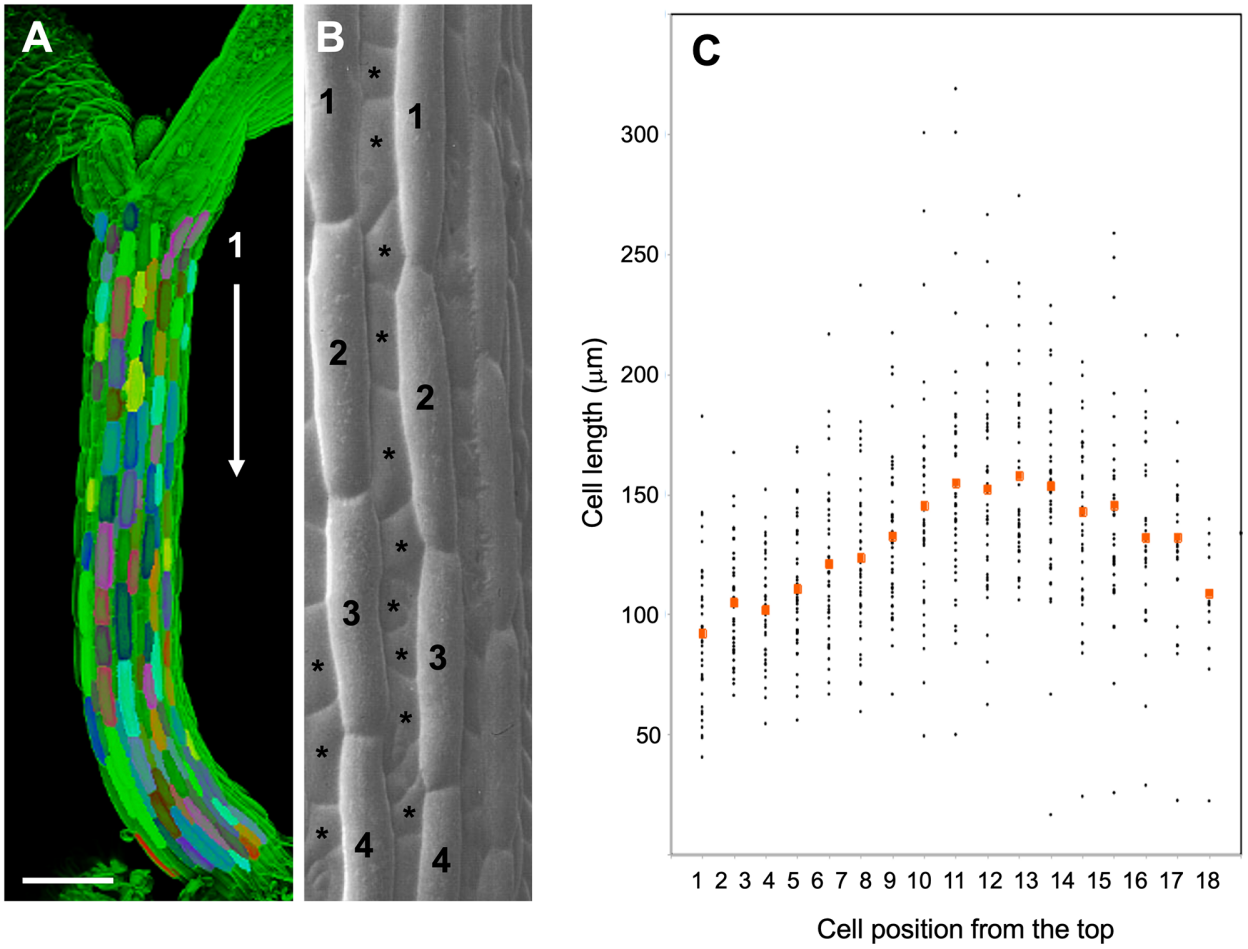
Cell length analysis in epidermal cell files of the Arabidopsis hypocotyl. **(A)** 3D-reconstruction from a confocal stack of an Arabidopsis hypocotyl 4 days after germination. Individual epidermal cells along the major cell files are highlighted in various colors. Size bar, 200 µm. **(B)** Scanning electron micrograph showing the two main cell types in the Arabidopsis hypocotyl epidermis. Major cell files (numbered cells), which alternate with minor cell files (asterisks), where used for cell length measurements. **(C)** Cell length of hypocotyl epidermal cells from top to bottom of Arabidopsis hypocotyls. Seedlings were grown for 5 days in long days under high light conditions (130µE). Red symbols indicate the mean cell size at the respective position from top (cell 1) to bottom (cell 18) of the hypocotyls. **(B)** was modified with permission from (Berger et al., 1998).

In order to address SAS at the cellular level, we first assessed the general growth patterns along the Arabidopsis hypocotyl by using the large epidermal cells (**Figures 1A, B**) as units to quantify local growth along the hypocotyl. As reported previously (Gendreau et al., 1997; Le et al., 2005), cellular growth was most prominent in the middle part of the hypocotyls, relative to the top and the bottom (**Figure 1C**). Since SAS is mediated by the growth hormone auxin (de Wit et al., 2016), a plausible explanation for such a growth pattern could be the distribution of auxin along the hypocotyl. In order to test this possibility, we employed an auxin reporter (DR5::GUS) that has been widely used in Arabidopsis (Ulmasov et al., 1997), including in the hypocotyl during directional growth in response to unilateral light or gravitropic stimulation (Friml et al., 2002). However, in our hands, DR5::GUS expression was below the detection limit under control conditions (high R:FR), as well as under low R:FR (**Supplementary Figure S1A**), although it was readily inducible by exogenous auxin (**Supplementary Figures S1B, C**). An improved auxin marker, DR5v2::ntdTomato (Liao et al., 2015), exhibited higher basal expression levels (**Supplementary Figure S1D**), however, it was not suitable for our purposes due to an irregular SAS response in the marker line (data not shown). We conclude that auxin levels in the hypocotyl are rather low, and that changes in auxin distribution elicited by low R:FR ratio under our conditions are too subtle to be detected by DR5-based reporters.

Considering that cell expansion is correlated with auxin concentration as in tropic growth responses (Friml et al., 2002; Christie et al., 2011), and that auxin drives hypocotyl growth in SAS (Tao et al., 2008; Keuskamp et al., 2010; Kohnen et al., 2016; Pucciariello et al., 2018), we assume that the cellular growth patterns in hypocotyls reflect shallow auxin gradients with maximal concentration differences in the range of 1.5-2.5-fold (highest vs. lowest values). Conceivably, such concentration gradients are less pronounced and shallower than the sharp local auxin maxima in root and shoot meristems (Benkova et al., 2003; Smith et al., 2006), providing an explanation for the failure of auxin reporters to reveal them (**Supplementary Figure S1**).

### Identifying cellular and molecular components for a mathematical model of SAS

In order to explore how the shade avoidance syndrome (SAS) could be controlled by shallow auxin gradients along the hypocotyl, we developed a mathematical model of the *Arabidopsis* hypocotyl. As a first step, we defined the essential molecular components that contribute to the phenomenon and which therefore need to be incorporated into the model. According to published evidence (Tao et al., 2008), SAS in seedlings is driven by auxin from the cotyledons that is transported towards the root by polar auxin transport (PAT) (de Wit et al., 2016). A reduced R:FR ratio in response to shading induces auxin biosynthesis in the cotyledons, resulting in elevated auxin levels along the hypocotyl, which promote cell expansion and tissue growth. Two transport systems mediate auxin transport through plant tissues, members of the PIN-FORMED (PIN) protein family, and members of the ATP-BINDING CASSETTE (ABC) transporter family, in particular subfamily B (ABCB) (Geisler, 2021). Based on functional analysis with knock-out mutants, the main auxin transporters in the regulation of hypocotyl growth are *PIN3*, *PIN4*, and *PIN7* (Keuskamp et al., 2010; Kohnen et al., 2016), as well as *ABCB1* and *ABCB19* (Wu et al., 2010, 2016, 2018). A third class of transporters, permease-like auxin influx carriers (AUX1/LAX), is important for many developmental phenomena, however, it has not been implicated in SAS (Swarup and Peret, 2012). In addition to transport across membranes, auxin diffusion through plasmodesmata contributes to the regulation of auxin distribution in the tissues (Han et al., 2014; Gao et al., 2020).

**Figure 2:**
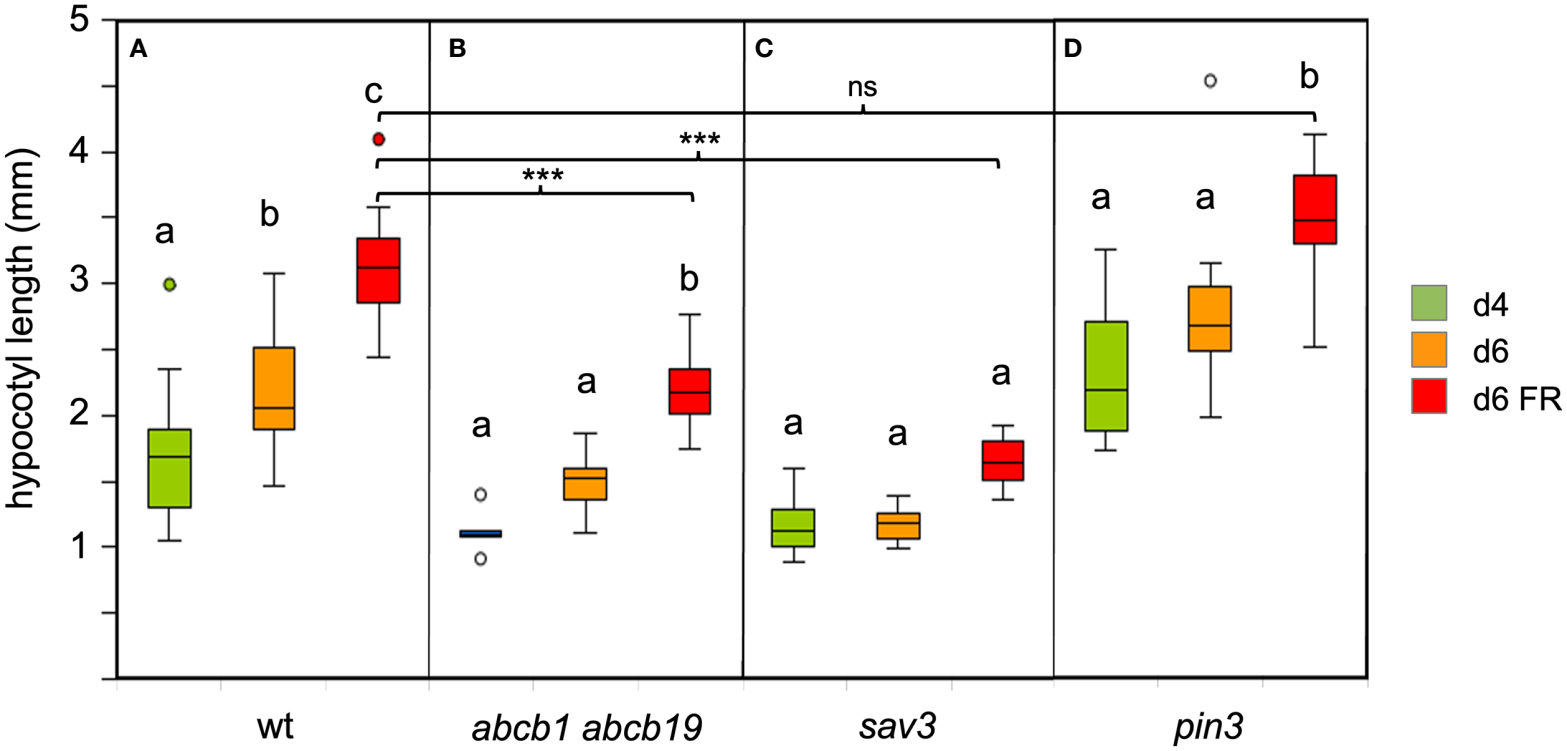
Growth response of *Arabidopsis* wild type **(A)** and several auxin-related mutants under simulated shade as indicated **(B–D)**. 4d-old seedlings (green boxes) were grown under CL75 exposed to high R:FR ratio simulating full day light (standard light, orange boxes), and to low R:FR ratio simulating shade (red boxes) for two days. Growth was affected in *abcb1 abcb19* and *sav3* mutants, but not in *pin3* mutants. Box plots represent the median and the interquartile range (IQR) of hypocotyl length with the whiskers indicating the minimal and maximal values within the range of 1.5 IQR above and below the box (n>6). Outliers are values beyond the whisker boundaries. Significant differences among treatments per genotype (p<0.05; two-way ANOVA and Tukey’s multiple comparison test) are indicated by letters; Significant differences among the FR-treated plants are indicated with asterisks; ns, non-significant (***<0.001; two-way ANOVA and Tukey’s multiple comparison test).

Among PIN proteins, the role of *PIN3* in hypocotyl growth is best documented (Friml et al., 2002; Keuskamp et al., 2010), and it is expressed at the highest levels in seedlings, however, all three PINs (*PIN3*, *PIN4*, and *PIN7*) act together in SAS in a partially redundant manner (Kohnen et al., 2016). *ABCB1* and *ABCB19* act redundantly, since mutants in either gene have very weak phenotypes, whereas the double mutant exhibits a severe growth phenotype (Geisler et al., 2003). Consistent with their redundant function, ABC1-GFP and ABC19-GFP have overlapping expression and localization patterns (Wang et al., 2013). These results suggest that cellular auxin transport in the hypocotyl encompasses two distinct components, a polar (PIN proteins), and a non-polar (ABCB proteins) mechanism. While the role of PIN proteins in SAS is well established (Keuskamp et al., 2010; Kohnen et al., 2016), the role of ABCB1 and ABCB19 in SAS is less clear. We found that the *abcb1 abcb19* double mutant was severely compromised in SAS (**Figure 2B**, compare with **Figure 2A**), suggesting that the non-polar component of auxin transport is as important in SAS as the polar component (Kohnen et al., 2016).

**Figure 3:**
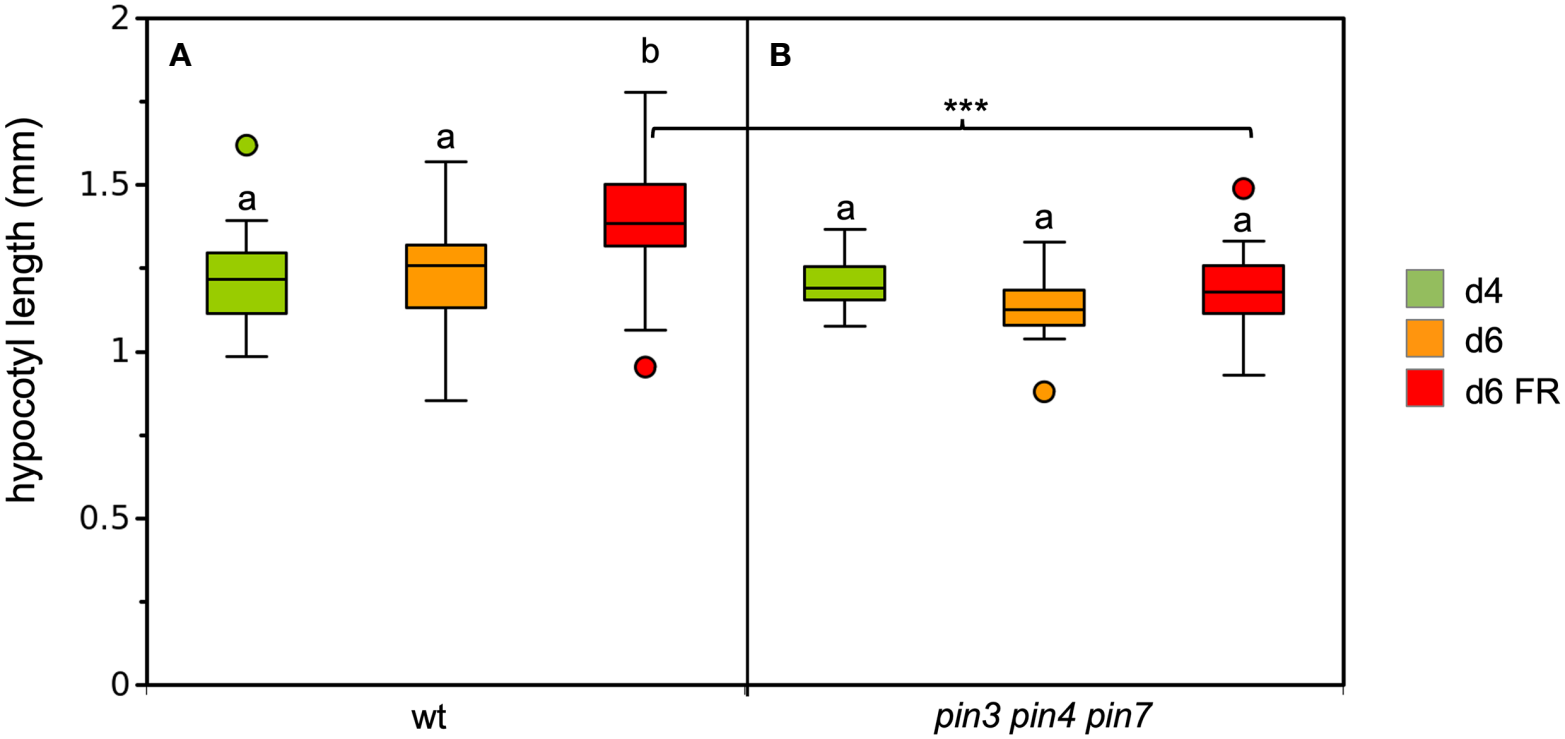
Growth response of the wild type **(A)**, and the *pin3 pin4 pin7* triple mutant under simulated shade **(B)**. 4d-old seedlings (green boxes) were grown under CL75 exposed to high R:FR ratio simulating full day light (orange boxes), and to low R:FR ratio simulating shade (red boxes) for one day. Box plots represent the median and the interquartile range (IQR) of hypocotyl length with the whiskers indicating the minimal and maximal values within the range of 1.5 IQR above and below the box (n>19). Outliers are values beyond the whisker boundaries. Significant differences among treatments per genotype (p<0.05; two-way ANOVA and Tukey’s multiple comparison test) are indicated by letters; Significant differences among the FR-treated plants are indicated with asterisks (***<0.001; two-way ANOVA and Tukey’s multiple comparison test).

In order to assess the dependence of SAS on auxin biosynthesis, we exposed the auxin biosynthetic mutant *sav3*/*taa1* (Tao et al., 2008) to low R:FR conditions. Consistent with previous reports (Tao et al., 2008; Kohnen et al., 2016), *sav3* mutants were severely compromised in their SAS response (**Figure 2C**). Surprisingly, *pin3* mutants did not show a defect in hypocotyl elongation (**Figure 2D**), in contrast to previous reports (Keuskamp et al., 2010). To verify a role of PAT in SAS, we used the *pin3 pin4 pin7* triple mutant for a growth assay under low R:FR ratio. Indeed, the triple mutants were inhibited in FR-dependent elongation growth (**Figure 3**), consistent with the assumption that PAT is required for SAS, and that PINs function at least partially redundantly (Kohnen et al., 2016). These results confirm the central elements of the SAS: auxin biosynthesis (involving SAV3/TAA1), polar auxin transport (involving PIN3, PIN4, and PIN7), and non-polar auxin transport (involving ABCB1 and ABCB19).

### Anatomical constraints for a mathematical model of SAS

**Figure 4:**
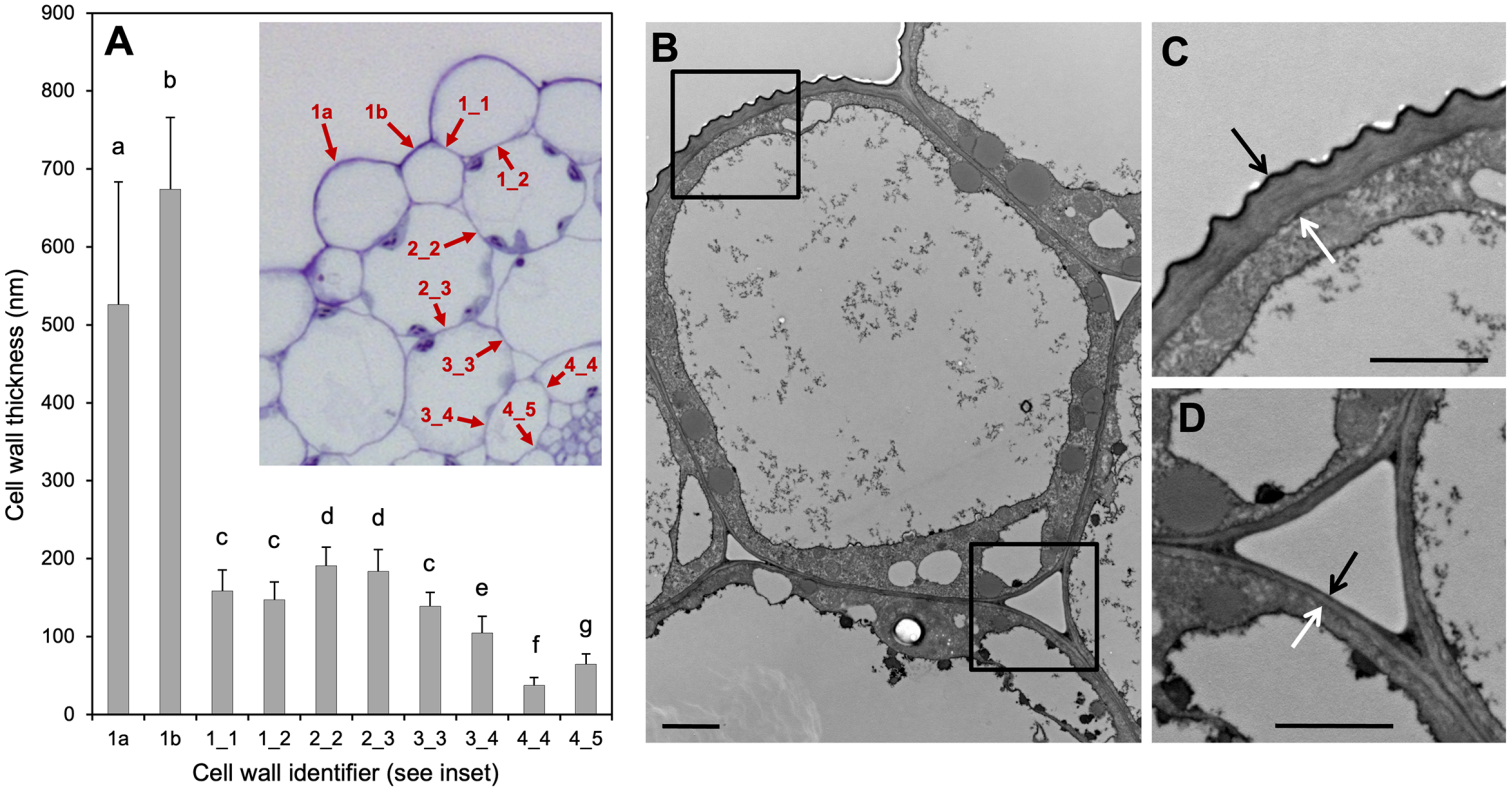
Cell wall thickness of hypocotyl cells. **(A)** Quantification of all cell walls in the Arabidopsis hypocotyl according to the inset in **(A)**. Cell wall thickness was measured from transmission electron micrographs as in **(B–D)**. Numbers in the inset refer to epidermis (1), outer cortex (2), inner cortex (3), and endodermis (4). Cell walls between two epidermis cells are referred to as 1_1, between epidermis and outer cortex as 1_2 etc., while 1a and 1b refer to major and minor cell files of the epidermis, respectively (see **(A)**, inset). Columns represent the mean +SD (n>40). Significant differences in cell wall thickness (p<0.05; one-way ANOVA and Tukey’s multiple comparison test) are indicated by different letters. **(B)** Major epidermal cell [1a in **(A)**]. **(C)** Close-up from **(B)** (upper left corner). **(D)** Close-up from **(B)** (lower right corner). Arrows in **(C, D)** indicate cell wall thickness.

An explicit growth model should take into account not only the molecular/genetic components, but also the anatomical conditions relevant for growth. In particular, it is important to know which cells limit organ growth, because these cells would be likely to control organ growth. In plant tissues, internal turgor pressure is thought to promote organ growth, while the cell wall limits growth and defines cell and organ shape (Kutschera and Niklas, 2013). We performed cell wall measurements on transmission electron micrographs to test which cell layer is likely to limit hypocotyl growth. We found that the outermost cell walls of the epidermal cell layers are much thicker than all the inner cell walls of all other cell types (**Figure 4**). In agreement with the conclusions derived from earlier genetic studies (Kutschera and Niklas, 2007; Savaldi-Goldstein et al., 2007), we therefore consider hypocotyl growth to be limited by the epidermis. This means that although all cells could potentially grow according to their auxin concentrations, only the auxin concentrations in epidermal cells are relevant, because growth potential of the entire organ along its long axis is restricted (and therefore dictated) by the epidermis.

### Concept of an auxin transport model for the Arabidopsis hypocotyl

**Figure 5:**
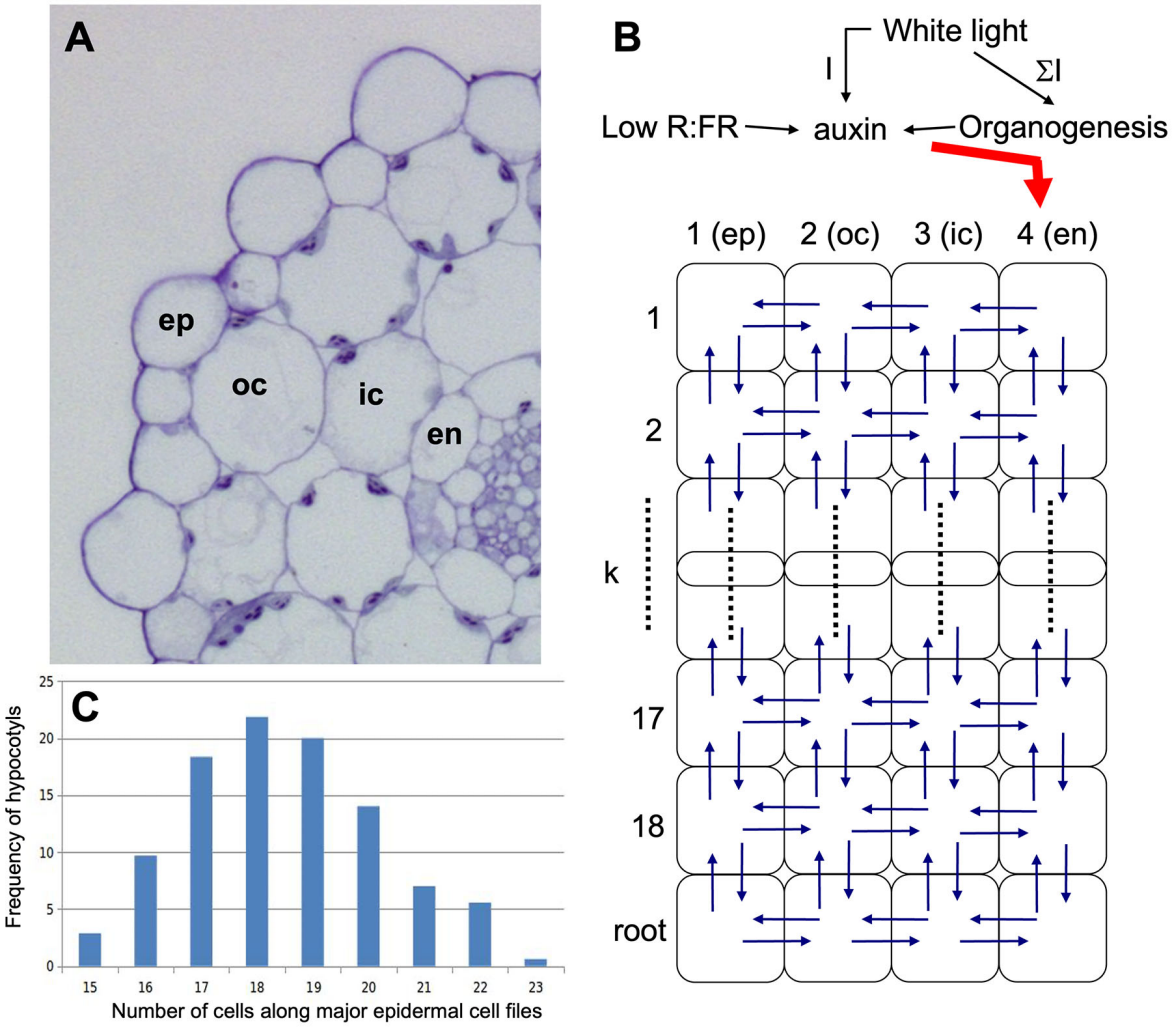
Structure of the cellular growth model for the Arabidopsis hypocotyl. **(A)** For determination of the cellular matrix of the model, the four external cell layers are considered: epidermis (ep), outer cortex (oc), inner cortex (ic), and endodermis (en). **(B)** Structure of the model. The hypocotyl is represented as a 2-dimensional matrix of four longitudinal cell layers with each 18 cells in length. A root compartment (represented as a single cell row) was added at the bottom to avoid boundary problems at the lower end of the hypocotyl. Auxin is produced in the cotyledons and enters the hypocotyl via the endodermis. Auxin biosynthesis is proportional to cotyledon size (represented by the sum of absorbed light). Low R:FR ratio stimulates auxin biosynthesis, whereas white light at elevated levels triggers conversion of auxin to an inactive form. **(C)** Epidermal cell number along the hypocotyl was between 15 and 23 cells per cell file, with the maximum at 18 cells, hence this value was chosen for the number of cell rows in the model.

In order to get insight into mechanisms that may control hypocotyl growth via shallow auxin gradients, we developed an auxin transport model that incorporates all the features mentioned above. The model assumes that hypocotyl growth is controlled by auxin distribution in the epidermis, which depends on four factors: (i) auxin production in the cotyledons and import through the endodermis, (ii) basipetal auxin transport along the endodermis (phloem transport may indirectly contribute, but would be relevant only after auxin passage through the endodermis), (iii) centrifugal auxin transport from the endodermis to the epidermis, and (iv) auxin catabolism along the transport path through the hypocotyl (**Figure 5**). For simplicity, the concentric organization of the hypocotyl with four cell layers, epidermis (ep), outer cortex (oc), inner cortex (ic), and endodermis (en) (**Figure 5A**) is represented in a single conceptual longitudinal section (**Figure 5B**). According with the average cell number observed in longitudinal cell files of the hypocotyls (**Figure 5C**), the conceptual hypocotyl has 18 cell rows in length (**Figure 5B**). Auxin biosynthesis in the cotyledons depends on the amount and quality of light, and on the amount of apical tissues (**Figure 5B**). SAS is modeled to influence hypocotyl growth by impinging on biosynthetic capacity in the cotyledons (**Figure 5B**). As a central feature, the model is based on established patterns of expression and localization of auxin transporters in the hypocotyl (Keuskamp et al., 2010; Wang et al., 2013). Our model is inspired by previous mathematical models of auxin transport in roots (Grieneisen et al., 2007), and in the shoot apex (Barbier de Reuille et al., 2006; Jönsson et al., 2006; Smith et al., 2006; Hartmann et al., 2019), however, as an important difference, we did not introduce a feedback by auxin onto its own transport. Most models generate sharp auxin peaks because of a positive feedback loop that results in the transport of auxin from cells with lower concentration towards cells with higher concentration (van Berkel et al., 2013; Cieslak et al., 2015, 2021). Hence, small local auxin elevations (resulting from random noise or from developmentally controlled programs) are transformed to characteristic developmental patterns. However, quantitative regulation of tissue growth is a fundamentally different phenomenon than shoot and root patterning, hence different solutions are required.

### General assumptions and equations of the auxin transport model

The following 15 assumptions, and the corresponding equations, constitute the auxin transport model. The corresponding parameters where estimated based on information from the literature, where available (**Supplementary Table S1**).

1.) Light is homogeneously spread around the plant thereby excluding phototropic effects, thus a radial section of the hypocotyl is sufficient to model elongation of the entire structure (**Figure 5B**).

2.) The hypocotyl has four concentric cell layers: From the outside to the inside, the epidermis (ep), the outer cortex (oc), the inner cortex (ic), and the endodermis (en). The innermost tissues (pericycle, stele) are not represented in the model, since they are thought to have minor roles in growth and auxin transport. Auxin transport through the phloem is not explicitly considered, although it may indirectly contribute to hypocotyl growth through transfer via the endodermis.

3.) The hypocotyl has 18 cell rows, i.e. each of the four cell layers is 18 cells long from top to bottom (**Figure 5B**) according to measured cell numbers along the hypocotyl (**Figure 5C**). At the lower end, an additional cell row has been added in the model to represent the root compartment (**Figure 5B**).

4.) There is no cell division during the period of simulation (Gendreau et al., 1997), hence, cell number remains constant.

5.) Auxin is considered to be the only growth factor.

6.) The epidermis restricts hypocotyl elongation (Kutschera and Niklas, 2007; Savaldi-Goldstein et al., 2007). As a consequence, growth of the hypocotyl is controlled by the epidermis.

7.) Auxin levels in the epidermis control cell expansion. Increased auxin levels promote cell elongation, while the inner tissue layers (cortex, endodermis) follow passively.

8.) Elongation of epidermal cells is regulated cell-autonomously by their auxin concentration. This phenomenon is described by Equation 1A:


(1A) $$\frac{dl_{k}}{dt}=\lambda \cdot S_{1}\left(A_{k,ep},\;u_{\mu }\right)$$

where $l_{k}$ represents cell length of cell k (cell width is assumed to be constant), λ is the growth factor, $A_{k,ep}$ is the concentration of auxin in the epidermal cell k , $u_{\mu }$ is the translational value and $S_{1}$ is the sigmoid function n°1 (see Equation 4 below).

Cell length develops over time as described by Equation 1B:


(1B) $$l_{k}\left(t+1\right)=l_{k}\left(t\right)+dl_{k}\;\;\;\text{with}\;\;\;l_{k}\left(t=0\right)=10\;\mu m$$

9.) Auxin input into the hypocotyl is proportional to auxin biosynthesis, which is considered to depend on light intensity and light quality (see Equations 2, 5). It follows a Monod relation over time proportional to the integral of light intensity over time (reflecting the relative size of the cotyledons as the auxin-producing tissue) (Ljung et al., 2001).

10.) Far red light (FR) intensity ( $I_{fr}$ ) stimulates auxin production over time, with a maximal production rate noted as μ (see Equation 2). Red light intensity ( $I_{r}$ ) is considered to be a constant as part of the direct white photosynthetically active radiation (PAR).

11.) FR is switched on at day 4. We defined the function $I_{fr}\left(t\right)$ to take into account the relative FR light intensity over time. According to Hennig et al. (1999), phytochrome declines with apparent first-order kinetics with a half-life of about 30 min but approximately ϵ (~30%) of total phytochrome remain active.

We obtain that


$$I_{fr}\left(t\right)\;=\;\{\begin{array}{cc} 1 & \text{in white-light condition}\\ I_{fr,max}\cdot \;2^{-2t} & \text{for}\;t\leq \;-log_{2}\left(\epsilon \right)/2\;\text{after switching on the FR}\\ \epsilon \cdot I_{fr,max} & \text{for}\;t>\;-log_{2}\left(\epsilon \right)/2\;\text{after switching on the FR} \end{array}\;$$

where $I_{fr,max}$ is the maximal FR light intensity parameter.

12.) A low $I_{r}/I_{fr}$ ratio results in an increase of auxin production (Halliday et al., 2009; Casal, 2012). The equation is described as follows:


(2) $$\frac{dq_{1,en}}{dt}=\mu \cdot \alpha \left(t\right)\cdot M_{1}\left(I_{c0}{+}_{\sum }^{t}I\left(t\right),u_{c}\right)$$

where $q_{1,en}$ is the quantity of auxin that enters the endodermal cell 1 (uppermost endodermal cell), μ  is the maximum quantity of input auxin, $I_{c0}$ is the initial light integration value (set arbitrarily to 25 μ E), I(t) is the light intensity at time t , $u_{c}$ is the translational value, and $M_{1}$ is a Monod function (see Equation 5 below).

The function α(t) describes the effect of R/FR ratio at time  t on auxin production. It follows this rule:


$$\alpha \left(t\right)=1-S_{2}\left(\frac{\text{β}}{I_{fr}\left(t\right)},u_{fr}\right)$$

with β  the natural $I_{r}/I_{fr}$ ratio (nRFR parameter), $I_{fr}\left(t\right)$ the relative FR light intensity, $u_{fr}$ is the translational value, and $S_{2}$ the sigmoid function n°2 (see Equation 4 below).

13.) All cells have the same constant auxin permeabilities, which, however, are different on the four sides of the cells (**Figure 5B**; Equation 3). Auxin can be imported or exported from/to neighbouring cells according to the arrows in **Figure 5B**.

14.) In a cellular network defined by the hypocotyl cell layers (ep-oc-ic-en) and rows (1 to 18), j ∼ i correspond to cell j neighbouring the cell  i according to tissue organization depicted in **Figure 5B**, the variation of auxin quantity in a cell i depends on the transport to/from neighbouring cells, and auxin inactivation which is proportional to auxin concentration and light intensity I(t) through activation of auxin-conjugating enzymes (Takase et al., 2003). Auxin transport and degradation/inactivation is described as:


(3) $$\frac{dq_{i}}{dt}={\;}_{\sum }^{j∼i}\left(P_{ji}\cdot q_{j}\left(t\right)-P_{ij}\cdot q_{i}\left(t\right)\right)-n\cdot \frac{q_{i}\left(t\right)}{l_{i}\left(t\right)}\cdot S_{3}\left(I\left(t\right),u_{I}\right)$$

(rate of auxin quantity change in cell i ) = (sum of auxin input and output in cell i  *from/to cell* j *) – (degradation of auxin in cell* i *)* where $q_{i}\;$ is the quantity of auxin in cell i at time t , $l_{i}\;$ the cell length at time t , $P_{ij}$ is the permeability of the membrane of the cell i adjacent to cell j , η is a degradation parameter, $u_{I}$ is the translational value for the light intensity, and $S_{3}$ is the sigmoid function n°3 (Equation 4) that decreases auxin degradation under low light conditions.

The permeability coefficient $P_{ij}$ represents both, diffusive and active transport components. The last cell row (n°19) is considered as the root system which is assumed to import from the bottom of the hypocotyl as much auxin as is exported.

15.) All parameters are constant over time (see **Supplementary Table S1**).

Regulatory functions used in the model are:


(4) $$S_{n}\left(x,u\right)=\frac{{\left(x-u\right)}^{2}}{a_{n}+{\left(x-u\right)}^{2}}$$

and


(5) $$M_{n}\left(x,u\right)=\frac{\left(x-u\right)}{b_{n}+\left(x-u\right)}$$

The parameters $a_{n}$ , $b_{n}$ and u were fitted.

The score value of the fitting system is described as:


(6) $$J=dt\cdot \sum_{k=1}^{18}\left|\frac{l_{k}-\gamma_{k}\;}{l_{k}}\right|$$

with $l_{k}$ , the mean epidermal cell length measured for layer k of the hypocotyl, and $\gamma_{k}$ the corresponding cell length of cell k resulting from model simulation.

### Calibration and validation of the model

**Figure 6:**
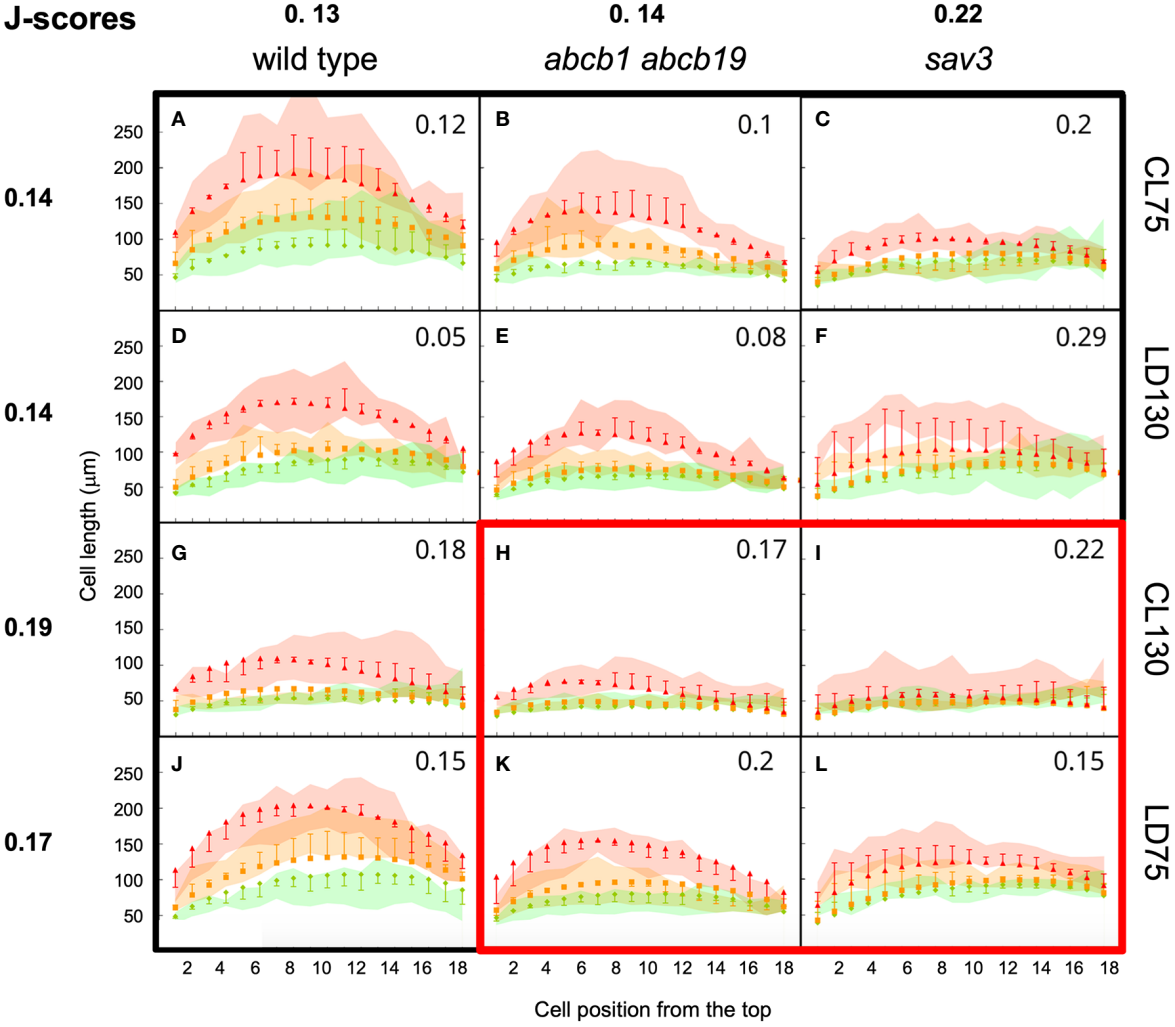
Calibration and validation of the growth model. Wild type plants **(A, D, G, J)**, *abcb1 abcb19* mutants **(B, E, H, K)** and *sav3* mutants **(C, F, I, L)** were grown at four different light conditions, continuous light (CL), and long day conditions (LD), each at a low and a high fluence rate (75 µE and 130 µE, respectively). Green lines, cell length at day 4; orange line, cell length at 6d under high R:FR conditions; red lines, cell length at 6d under low R:FR conditions. Colored surfaces encompass the 95% confidence interval. Squares, triangles, and diamonds represent the respective simulated cell length values with the whiskers representing the deviation of simulated values from experimental data points. **(A-F, G, J)** were used for parameter fitting, **(H, I, K, L)** were used for model validation. The characteristic arch-shaped size distribution is observed in all treatments and conditions, with decreased values in the mutants (*abcb1 abcb19* and *sav3*), and inhibition of growth at high light conditions (LD130, CL130). J-scores are indicated in each individual panel, and average J-scores were calculated for each genotype and condition.

In order to calibrate and validate the model, and to estimate optimal parameter values, we used experimental data from the wild type and the two mutants *abcb1 abcb19* and *sav3*, and from four different light conditions, continuous light (CL), and long day conditions (LD), each at a low and a high fluence rate (75 µE and 130 µE, respectively) (**Figure 6**). The strongest SAS was observed under continuous low light conditions (**Figures 6A–C**; **Supplementary Figure S2**), with clearly reduced responses of the mutants, in particular of *sav3* (**Supplementary Figure S2**). In general, high light caused weaker SAS than low light (**Figures 6D–I**; compare with **Figures 6A–C, J–L**).

A set of initial parameter values for parameter fitting was defined based on available information from the literature, or set to arbitrary values (**Supplementary Table S1**). We used part of the experimental data, namely continuous light at low fluence rate (CL75; **Figures 6A–C**), long day at high fluence rate (LD130; **Figures 6D–F**), and all wild type data (**Figures 6A, D, G, J**) for parameter fitting. The remaining data (**Figures 6H, I, K, L**) was used for model validation.

In general, the model recapitulated the growth patterns of Arabidopsis hypocotyls in three central aspects: i.) Firstly, growth was more prominent in the wild type (**Figures 6A, D, G, J**) than in the *abcb1 abcb19* double mutant (**Figures 6B, E, H, K**), and even weaker growth was observed in the *sav3* mutant (**Figures 6C, F, I, L**); ii.) secondly, growth was stronger under low light conditions (fluence rate of 75 µE; **Figures 6A–C, J–L**) than under high light (fluence rate of 130 µE; **Figures 6D–I**); iii.) and thirdly, the cellular growth pattern exhibited the typical arch-shaped distribution with longest cells in the middle of the hypocotyl (**Figure 6A**, compare with **Figure 1**).

**Figure 7:**
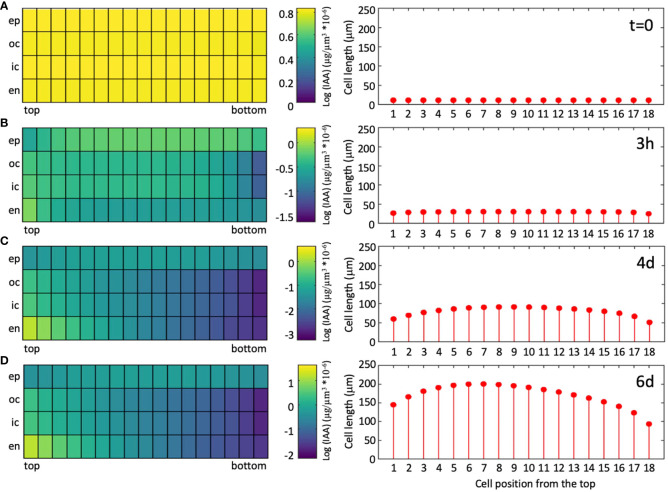
Simulated auxin distribution and cellular growth pattern in a wild-type hypocotyl undergoing SAS. Stills of movie S1 were extracted at the beginning of the growth period [**(A)**; t=0], after growth in high R/FR conditions for 3h **(B)**, and 4d **(C)**, and after an additional two days of simulated shade [**(D)**; low R/FR conditions].

**Figure 8:**
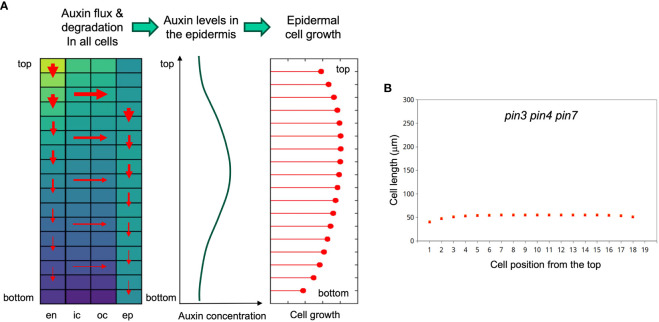
Principle of the growth model and simulation of the *pin3 pin4 pin7* triple mutant phenotype. **(A)** Left panel: Cellular matrix of the simulated hypocotyl with relative auxin concentrations of all cells indicated by the heat map (yellow high, blue low); red arrows indicating local net auxin fluxes, with thickness of the arrows reflecting flux strength (depending on local auxin sources and sinks, and on auxin degradation). Middle panel: These fluxes result in a flat auxin gradient in the epidermis, which determines growth potential in the epidermis (right panel). Epidermal growth potential dictates cell elongation patterns of the entire hypocotyl, because the cell layers are physically coupled. Therefore, all cell layers (epidermis, outer cortex, inner cortex, endodermis) grow at the same pace. **(B)** Simulation of SAS in the *pin3 pin4 pin7* triple mutant. Auxin import in the hypocotyl and PAT activity were reduced to 10% of the wild type. Note that there are three series of symbols (green diamonds, orange squares, red triangles), reflecting 4d continuous low light, and 2 days at high or low R/FR illumination, respectively (as in **Figure 6**). However, due to a complete overlay, the symbols of the three treatments cannot be distinguished. Hence, the simulated triple mutant does not grow at all under these conditions.

As a measure for the quality of the simulations, we calculated J-scores for each combination of genotype and condition, based on the deviations between measured and simulated values (Equation 6). Averaged values per genotype of the wild type (0.13), and of the *abcb1 abcb19* double mutant (0.14) were considerably better than for the *sav3* mutant (0.22), because in the latter case, plants grew more than in the simulations. This may reflect compensatory mechanisms in auxin biosynthesis (Zhao, 2010), that do not exist for auxin transport. Averaging the J-scores per growth conditions resulted in more similar values (between 0.14 and 0.19), indicating that the response to the growth conditions is represented well by the model. As expected, the average J-score of the data used for parameter fitting (**Figure 6A–G, J**; J-score=0.15), was lower than the J-score of the data used for validation (**Figures 6H, I, K, L**; J-score=0.19), however, the moderate difference reveals that the model has a decent predictive potential. Assessing the dynamics of auxin distribution and growth (**Supplementary Movie S1**; **Figure 7**) showed that a characteristic gradient with a flat peak around the central part of the hypocotyl is quickly established (**Figures 7A, B**), and remains stable for days (**Figures 7C, D**). Given the entry of auxin through the endodermis, basipetal polar transport in all cells, and a net outward component (due to the source in the endodermis), in conjunction with degradation along the entire transport path, results in an auxin distribution in the epidermis with highest levels in the middle part, which translates into the corresponding cellular growth pattern (**Figure 8A**).

Finally, we interrogated the model for the effects of mutation of the polar transport component. We simulated a *pin3 pin4 pin7* triple mutant by reducing the entry of auxin into the endodermis to 10% (considering that flux from the sites of biosynthesis in the cotyledons also requires PAT), and the polar transport component along the hypocotyl to 10% as well. This resulted in a complete block of growth in both light conditions (**Figure 8B**), consistent with the complete block of hypocotyl elongation in the *pin3 pin4 pin7* triple mutant, that was considerably stronger than growth inhibition in the *sav3* mutant (Kohnen et al., 2016).

Taken together, our mathematical model can reproduce the SAS in the *Arabidopsis* hypocotyl with remarkable predictive power. The combination of basipetal and centrifugal auxin transport, in conjunction with auxin degradation along the hypocotyl, generates shallow auxin gradients in the epidermis, that dictate the cellular growth pattern along the hypocotyl due to their thick growth-limiting external cell walls. As a consequence, the entire hypocotyl shows the characteristic growth dynamics in SAS with strongest growth in the middle part.

## Discussion

### The *Arabidopsis* hypocotyl as a growth model

One of the best-characterized experimental systems for growth in plants, the hypocotyl of *Arabidopsis thaliana*, grows almost exclusively by cell expansion (Gendreau et al., 1997). Furthermore, the hypocotyl exhibits essentially unidimensional elongation along the long axis of the tubular structure, which greatly facilitates the quantification of growth. Depending on the environmental conditions, different hormones have been implicated in regulation of hypocotyl elongation (de Wit et al., 2016; Yang and Li, 2017). However, in the case of SAS, the growth phenomenon is primarily controlled by auxin (de Wit et al., 2016).

Many growth processes from early embryogenesis to flowering are regulated by auxin (Davies, 2004). In the case of differential growth phenomena, such as during organogenesis (Benkova et al., 2003; Reinhardt et al., 2003) or tropic growth responses (Strohm et al., 2012; Fankhauser and Christie, 2015), polar auxin transport is thought to determine auxin distribution and thereby differential cell growth and cell fate. In Arabidopsis, auxin regulates hypocotyl elongation in the light (Jensen et al., 1998). Similarly, auxin mediates SAS, evidenced by mutants that are affected in auxin biosynthesis (Stepanova et al., 2008; Tao et al., 2008; Nozue et al., 2015), auxin transport (Keuskamp et al., 2010), auxin homeostasis (Zheng et al., 2016), or auxin-related signaling (Steindler et al., 1999; Hornitschek et al., 2012), and which all have strong defects in SAS.

### Shade avoidance, auxin, and cellular growth pattern of the *Arabidopsis* hypocotyl

Based on numerous studies, a current model of SAS posits that low R:FR ration is perceived in the cotyledons by phytochrome, resulting in increased auxin biosynthesis and transport of auxin into the hypocotyl by polar auxin transport where it accumulates and stimulates elongation growth (Casal, 2013; de Wit et al., 2016). Recently identified additional factors include the controlled conjugation of auxin and possibly the release of auxin from conjugated forms by hydrolysis (Zheng et al., 2016). However, apart from the regulation of SAS by auxin, the cellular growth patterns along the hypocotyl have not been addressed. While we are aware of the fact that other hormones also influence SAS, and hypocotyl growth in general (de Wit et al., 2016; Yang and Li, 2017), we focus here on auxin as the central regulatory principle.

In SAS, cell expansion along the hypocotyl is not uniform. Cells in the middle grow more than cells at the top and the bottom of the hypocotyl (Gendreau et al., 1997; Le et al., 2005; Robinson et al., 2017) (**Figure 1**). The basis of this cellular growth pattern is unknown, but it may be related to auxin distribution along the hypocotyl. Conceivably, auxin accumulates to higher levels in the middle of the hypocotyl than in the tip or the base, although the expected shallow auxin gradients could not be verified with available genetic marker tools.

### Auxin transport in SAS

Unexpectedly, *pin3* mutants did not exhibit a significant defect in SAS (**Figure 2**), however, the triple mutant *pin3 pin4 pin7* was strongly impaired in SAS (**Figure 3**) as described previously (Kohnen et al., 2016). In contrast to *pin3*, the *abcb1 abcb19* double mutant exhibited a pronounced defect in SAS (**Figure 2**). Taken together, these results suggest that SAS involves both, polar auxin transport via PIN proteins, and non-polar auxin transport by ABCB proteins, while the individual role of PIN3 is unclear, based on our results. These findings prompted us to ask, whether shallow auxin gradients could be formed in a cellular network as in the hypocotyl with a mechanism that does not require subcellular PIN protein re-localization. Indeed, a constitutive basipetal component (PIN proteins), and a non-polar transport mechanism (ABCB proteins), in conjunction with auxin metabolism (catabolism and/or auxin conjugation) is sufficient to generate auxin gradients that are in agreement with observed cellular growth patterns (**Figures 6**–**8**). Notably, such gradients emerge in the absence of subcellular PIN protein re-localization in response to SAS.

Importantly, such a mechanism would uncouple SAS, which has no inherent lateral growth component, from tropic growth mechanisms (phototropism, gravitropism), which involve polar reorientation of PIN proteins (Liscum et al., 2014). An SAS mechanism that involves PIN reorientation would require precise coordination around the hypocotyl to avoid hypocotyls to accidentally bend towards one side upon unequal auxin reallocation. Thus, plants would either require a precise radial coordination mechanism to ensure equilateral reorientation of PINs (and consequently auxin) around the circumference of the hypocotyl, or SAS would risk to interfere with tropic growth mechanisms. This is because uneven growth in response to SAS (i.e. bending of the hypocotyl) due to unequal reallocation of PINs (and auxin) around the circumference of the hypocotyl would need to be continuously corrected by gravitropism.

### Parameter sensitivity of the auxin transport model

**Figure 9:**
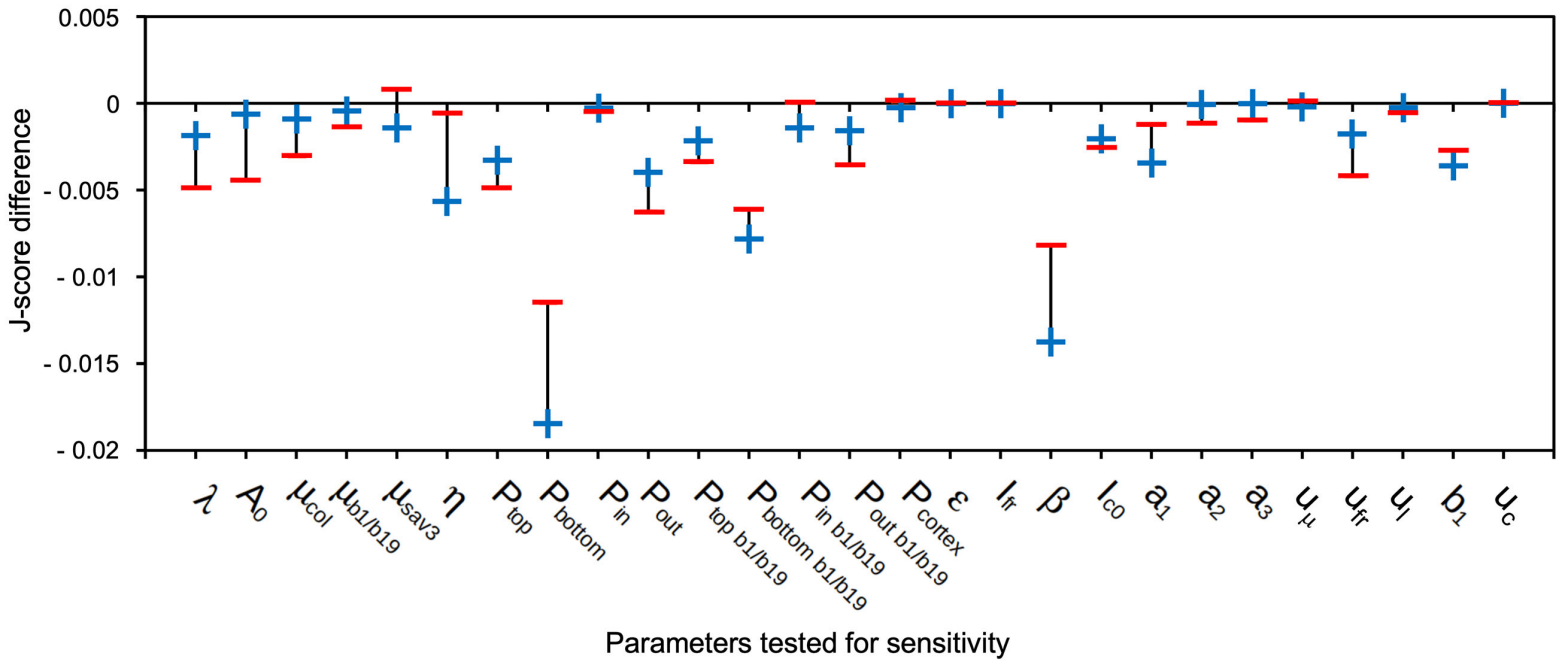
Sensitivity test for parameters of the auxin transport model. The model was tested for its sensitivity towards changes of parameters by increasing (+) and decreasing (-) them by 1%. Plotted is the J-score difference, i.e. (original global J-score) – (global J-score after 1% positive (+) or negative (-) change of the respective parameter). Global J-scores were calculated for each parameter over the entire set of conditions and genotypes used for fitting (**Figures 6A-G**, **J**) relative to the simulation with the unchanged parameters. Negative values in **Figure 9** indicate that global J-scores after the 1% change were increased (i.e. larger deviation from measured values than with the original parameters). The lower the negative values, the stronger the sensitivity of the parameter.

A mathematical model can enable predictions about regulatory mechanisms, if it reveals features that are robust and sensitive, respectively, towards endogenous or exogenous disturbances. Parameter sensitivity tests can reveal such potential nodes of regulation. In order to perform such a sensitivity test, we varied each parameter by 1% up or down, relative to the fitted values (see **Supplementary Table S1**). Subsequent simulations on the conditions used for parameter fitting (**Figures 6A–G, J**) revealed how much the newly obtained values for cell length deviated from the previous values (obtained with unchanged parameters). A global J-score was calculated for the two simulations resulting from the +1% deviation (+) and the -1% deviation (-) relative to the unchanged parameter (**Figure 9**). The strongest reduction in J-score quality was observed for the permeability at the bottom of hypocotyl cells (*P_bottom_*), reflecting polar basipetal auxin transport (**Figure 9**). The second-most sensitive parameter was β (the natural $I_{r}/I_{fr}$ ratio) (**Figure 9**), which is involved in the auxin production term (see above). These results of the sensitivity test indicate that, according to our model, the most sensitive aspects of SAS are i.) polar auxin transport, and, ii.) FR-induced auxin production, consistent with the dramatic SAS defect of the *pin3 pin4 pin7* triple mutant (**Figure 8B**) (Kohnen et al., 2016), and the *sav3* mutant (**Figure 6**) (Tao et al., 2008), respectively.

### How can auxin transport produce shallow gradients?

A central feature of many auxin transport models is a positive feedback loop in which auxin transporters become localized towards neighboring cells with higher auxin levels (Cieslak et al., 2021). This results in progressive accumulation of auxin in small cell populations in phyllotaxis (Shi and Vernoux, 2019; Reinhardt and Gola, 2022), or along narrow cell files in vascular patterning (Scarpella et al., 2006; Kneuper et al., 2021). While such positive feedback mechanisms can generate patterns that resemble natural patterns of growth and differentiation, it is unclear how adjacent cells could be mechanistically coupled. For example, how can a cell “know” the auxin concentration of its neighbors, in order to orient its own PIN auxin exporters towards the cell with the highest auxin concentration? Despite many open questions, most models agree with the assumption that such positive feedback loops are required for many patterning phenomena observed in plant development.

In contrast, such feedback mechanisms are unsuitable to generate shallow auxin gradients, as they would be expected to occur in cases of quantitative growth phenomena as in the hypocotyl. This is because small concentration differences inevitably become amplified to sharp narrow peaks by positive feedback mechanisms (Barbier de Reuille et al., 2006; Jönsson et al., 2006; Smith et al., 2006). Our transport model does not involve a positive feedback mechanism, instead, auxin distribution is determined by a combination of synthesis, two-dimensional transport (basipetal and centrifugal), and auxin catabolism along its transport route (**Figures 7**, **8A**, **Supplementary Movie S1**). All these aspects of auxin-dependent growth in hypocotyls are well supported by experimental evidence. Our model can generate shallow auxin gradients that could mediate quantitative tissue growth in hypocotyls (**Figure 8A**), and other tissues. In that respect, our model is conceptionally similar to models that generate morphogen gradients based on synthesis, diffusion, and spatially uniform degradation (SDD models) in animal systems (Grimm et al., 2010). While the model in its present form is designed to account for unidirectional elongation growth in the cylindrical hypocotyl, its principles can also be incorporated into advanced models of morphogenesis and tissue patterning in plants, or in anisotropic growth in response to exogenous cues, such as light or gravity in phototropism and gravitropism. Furthermore, future versions of our model can encompass additional levels of regulation, including gene expression, protein levels, subcellular protein dynamics, and cell division.

## Materials and methods

### Plant materials and treatments

*Arabidopsis thaliana* (L.) Heynh. accession Columbia (Col-0), *pin3-5* from NASC (N9364), *abcb1-101 abcb19-3* (Wu et al., 2016), *sav3-2* (Tao et al., 2008) and the *pin3-3 pin4-101 pin7-102* triple mutants (Willige et al., 2013; Kohnen et al., 2016) were germinated and grown as described (Reinhardt et al., 2003). The seeds were spaced by 5 mm in pots containing seedling substrate (Klasmann-Deilmann GmbH; https://klasmann-deilmann.com) and stratified at 4°C in the dark for two days. Then, seedlings were transferred to light (fluorescent tubes, Sylvania 36 W Luxline-Plus; https://www.sylvania-lighting.com) in a thermo- and hygro-regulated growth chamber (22 ± 1°C and 73 ± 2% rH), which defined the t=0 time point of the germination process. Seedlings were grown in high light (130 mmol m^−2^ s^−1^ PAR) or low light (75 mmol m^−2^ s^−1^ PAR) in continuous light (CL) or long day photoperiod (L:D, 16 h:8 h) for 6 days. Far-Red light was added at 4 days with a led lamp (LumiBulb-FR LED Growth Bulb, LumiGrow, USA; https://www.lumigrow.com) placed on top at a distance of 16 cm from the seedlings.

### Cell length analysis

Seedlings were collected at indicated time points (4, 5, or 6 days after germination), incubated in 0.5 M KCl solution for 4 h and then in Calcofluor white (0.1%, Tris-HCl 0.1 M, pH=8.5, Merck) for 24 h. Cell length was measured with ImageJ 1.46a (NIH) from image stacks acquired with a laser scanning confocal microscopy (Leica, TCS SP5). Cell length was determined for all cells (cell 1-18 from the top) in three representative epidermal cell rows along the hypocotyls.

### Measurement of cell wall thickness

Seedlings were fixed in 50 mM Na-cacodylate buffer pH =7.4 with 2% (v/v) glutaraldehyde (EMS; http://www.ems-group.com) for 2h at room temperature. After six washes with 50 mM Na-cacodylate buffer pH=7.4, the samples were postfixed overnight with 1% (w/v) OsO_4_ in Na-cacodylate buffer pH=7.4 at 4°C. After six washes in cacodylate buffer and one final wash with water, the samples were dehydrated through an acetone series (10%, 20%, 30%, 50%, 70%, 90%, 100%, each 10 min), and seven subsequent changes with acetone. Embedding proceeded with serial incubation in increasing concentrations of Spurr’s resin (Plano; https://www.plano-em.de) in acetone (25% 1.5h, 50% 1.5h, 75% overnight, 100% 6 h), and polymerization at 70°C for 18 h under dry atmosphere (silicagel). Ultrathin sections (70 nm) were prepared with a Reichert Ultracut E microtome (Leica microsystems) and mounted on formvar-coated grids. Sections were contrasted with 2% (w/v) uranyl acetate and subsequently with 80 mM lead citrate (Reynolds, 1963). Electron micrographs were taken with a Philips CM 100 BIOTWIN electron microscope (FEI Company, Eindhoven, The Netherlands) at 80 kV using a LaB6 cathode and a 11 MegaPixel TEM CCD Camera from Morada (EMSIS, Germany). Cell wall thickness was determined with the software iTEM Soft Imaging System. Multiple measurements were taken from several cells per section to calculate the average cell wall width. For each treatment several sections were taken from two replicate plants.

### Staining and imaging of DR5::GUS seedlings

Whole seedlings were fixed with acetone (90%) for 20min at room temperature after exposure with IAA at the indicated concentrations for 24 h. Then, the seedlings were infiltrated with staining buffer for 10 min on ice. Staining buffer consisted of 100mM sodium phosphate at pH 7.2, 10 mM EDTA, 0.2% triton X-100, 1 mM Ferricyanide, 1 mM Ferrocyanide and H_2_O (96%). This was followed by a second infiltration of 20 min on ice with 2mM X-GlcA cyclohexyl ammonium salt added to fresh staining buffer. Samples were then transferred to 37°C for 24 hours. Samples were then washed with increasing ethanol concentrations of 25, 50, 75 and 95% for 20min each. Samples were stored at 4°C in ethanol until imaging. Images were taken with a Leica DMR microscope equipped with a Zeiss Axiocam.

### Auxin flux model

The model is based on the assumption that auxin enters the hypocotyl by the top cell of the endodermis, and then is transported basipetally and centrifugally towards the epidermis. At the same time, auxin undergoes inactivation (corresponding to degradation or conjugation). Cell growth is assumed to be proportional to cellular auxin concentration. Initial parameters were derived from the literature, or defined arbitrarily, and optimized by the “simulated annealing” method (Kirkpatrick et al., 1983) to fit the measured cell length data. In order to reduce the computational time, original parameters were estimated by an approximation approach, then, parameters were optimized twice. The open-source software GNU Octave and Matlab (Natick, Massachusetts: The MathWorks Inc.; https://www.mathworks.com) were used for parameter optimization as described (Feller et al., 2015).

## Data availability statement

The original contributions presented in the study are included in the article/**Supplementary Material**. Further inquiries can be directed to the corresponding author.

## Author contributions

PF: Conceptualization, Data curation, Formal Analysis, Investigation, Software, Writing – original draft, Writing – review & editing. Ev: Conceptualization, Data curation, Investigation, Writing – review & editing. MS: Data curation, Formal Analysis, Investigation, Methodology, Visualization, Writing – original draft. FY: Conceptualization, Formal Analysis, Investigation, Methodology, Software, Writing – original draft. DR: Conceptualization, Funding acquisition, Investigation, Project administration, Supervision, Validation, Visualization, Writing – original draft, Writing – review & editing.

## Supplementary material

Data Sheet 2 Mathematical code and various associated files.

Supplementary Image 1 Evaluation of DR5-based auxin markers. **(A)** DR5::GUS was not sufficiently sensitive to detect auxin in the hypocotyl under our growth conditions. Nevertheless, DR5::GUS was inducible by treatment with the auxin indole-3-acetic acid (IAA) at 10 µM **(B)**, or 100 µM **(C)**, respectively, for 24 h in aqueous solution (applied from a 1000x stock in DMSO). **(D)** DR5v2::ntdTomato was sufficiently sensitive to detect auxin in hypocotyls of the reporter line DR5v2::ntdTomato/DR5::n3GFP, however, no changes were observed in our experiments, indicating that subtle changes in auxin concentration cannot be detected with this tool. Signal from ntdTomato was acquired in the red channel but is represented in false green; autofluorescence in the far-red range (highlighting mainly chlorophyll) was acquired to visualize the structure and outline of the hypocotyl.

Supplementary Image 2 Cell size distribution in the hypocotyls of various genotypes. Wild type plants **(A)**, *abcb1 abcb19* mutants **(B)**, and *sav3* mutants **(C)** were subjected to light conditions as in **Figure 6A–C**. Colored dots in **(A)** indicate significant differences of the low R:FR treatment (green line) vs. high R:FR treatment (red line). Red dots, p<0.001; orange dots, p<0.01; yellow dots, p<0.05 (n>20). Colored dots in **(B, C)** indicate significant differences between the respective mutant at low R:FR treatment (green lines in **(B, C)**) vs. the low R:FR treatment in the wild type (green line in **(A)**). Red dots, p<0.001; orange dots, p<0.01; yellow dots, p<0.05 (n>20).

Supplementary Image 3 Experimental design. Seeds were sown and stratified in the dark at 4°C for two days. Seedlings were cultured in white-light at 22°C (continuous light or long-days light). After four days in white light, seedlings received FR enrichment for two days (except for control seedlings). Seedlings were sampled at four days and six days for analysis.

Video 1 Visual rendering of a simulation of cellular patterns of auxin concentration and cell growth in a wild-type hypocotyl growing under continuous low white light for 4 days, followed by 2 days of simulated shade with low R/FR ratio compare with **Figure 6A**, and **Figure 7**.

Supplementary Table 1 List of parameters used in the growth model.
