# Supplementary figures and images for: Regulation of tissue growth in plants – A mathematical modeling study on shade avoidance response in Arabidopsis hypocotyls
Source: Front Plant Sci. 2024 Feb 28;15:1285655. doi: 10.3389/fpls.2024.1285655 (PMC10938469; doi:10.3389/fpls.2024.1285655)

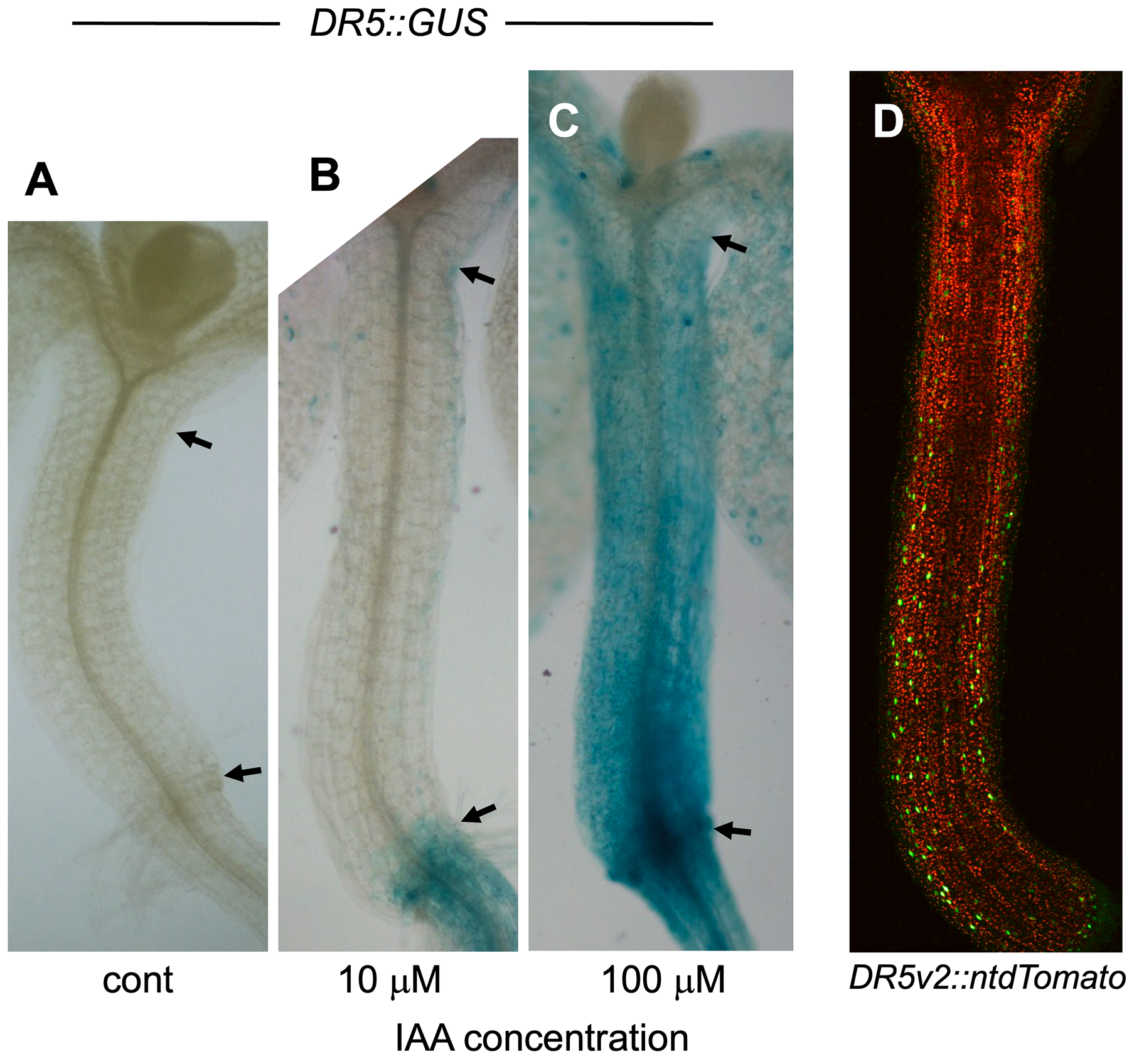

Supplement: Supplementary Image 1 — Evaluation of DR5-based auxin markers. (A) DR5::GUS was not sufficiently sensitive to detect auxin in the hypocotyl under our growth conditions. Nevertheless, DR5::GUS was inducible by treatment with the auxin indole-3-acetic acid (IAA) at 10 µM (B), or 100 µM (C), respectively, for 24 h in aqueous solution (applied from a 1000x stock in DMSO). (D) DR5v2::ntdTomato was sufficiently sensitive to detect auxin in hypocotyls of the reporter line DR5v2::ntdTomato/DR5::n3GFP, however, no changes were observed in our experiments, indicating that subtle changes in auxin concentration cannot be detected with this tool. Signal from ntdTomato was acquired in the red channel but is represented in false green; autofluorescence in the far-red range (highlighting mainly chlorophyll) was acquired to visualize the structure and outline of the hypocotyl. [file Image_1.tif]

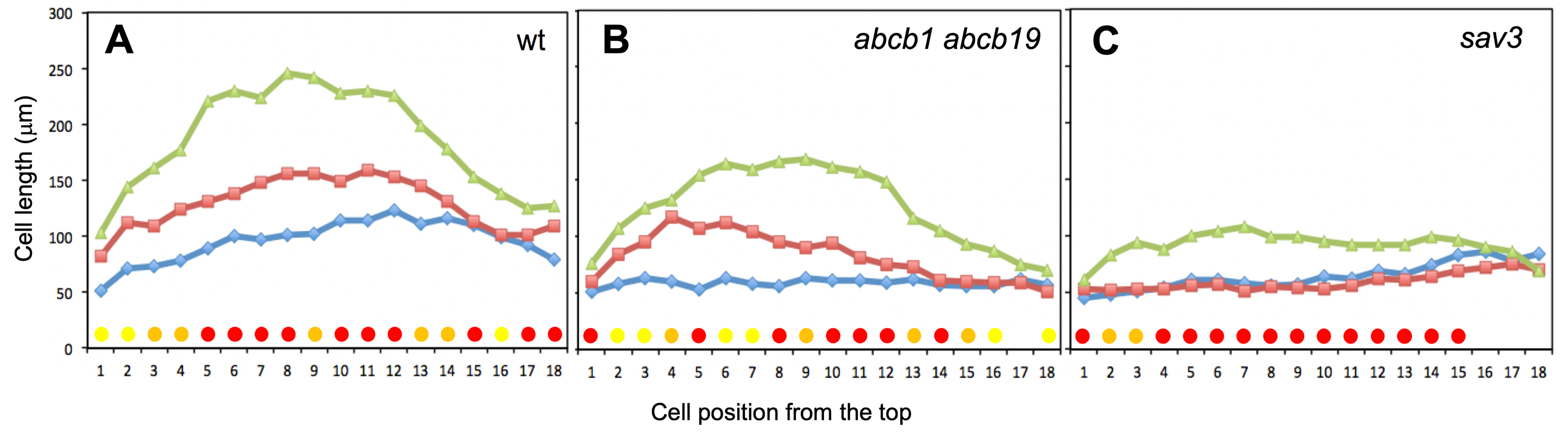

Supplement: Supplementary Image 2 — Cell size distribution in the hypocotyls of various genotypes. Wild type plants (A), abcb1 abcb19 mutants (B), and sav3 mutants (C) were subjected to light conditions as in Figure 6A–C . Colored dots in (A) indicate significant differences of the low R:FR treatment (green line) vs. high R:FR treatment (red line). Red dots, p<0.001; orange dots, p<0.01; yellow dots, p<0.05 (n>20). Colored dots in (B, C) indicate significant differences between the respective mutant at low R:FR treatment (green lines in (B, C)) vs. the low R:FR treatment in the wild type (green line in (A)). Red dots, p<0.001; orange dots, p<0.01; yellow dots, p<0.05 (n>20). [file Image_2.tif]

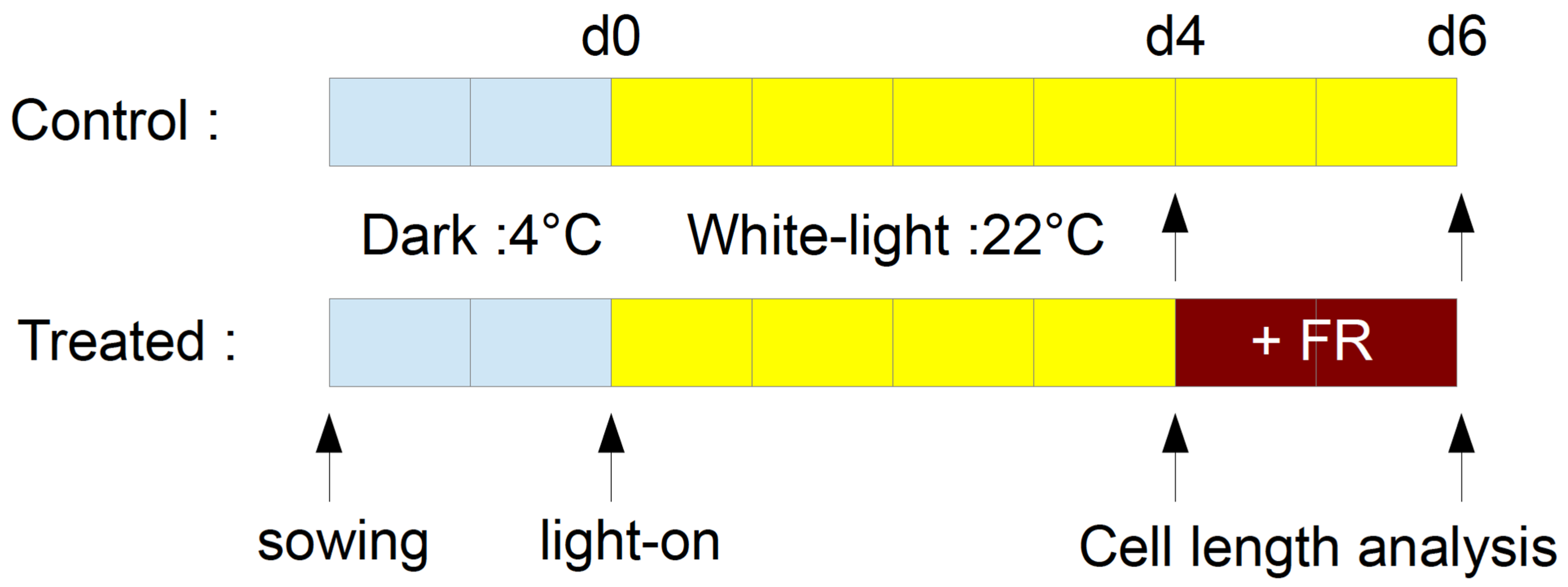

Supplement: Supplementary Image 3 — Experimental design. Seeds were sown and stratified in the dark at 4°C for two days. Seedlings were cultured in white-light at 22°C (continuous light or long-days light). After four days in white light, seedlings received FR enrichment for two days (except for control seedlings). Seedlings were sampled at four days and six days for analysis. [file Image_3.tif]
