# Supplementary material for: Regulation of tissue growth in plants – A mathematical modeling study on shade avoidance response in Arabidopsis hypocotyls
Source: Front Plant Sci. 2024 Feb 28;15:1285655. doi: 10.3389/fpls.2024.1285655 (PMC10938469; doi:10.3389/fpls.2024.1285655)
Supplement: Supplementary Table 1 — List of parameters used in the growth model. [file Table_1.pdf]

Table S1. Parameters and related information of the hypocotyl growth model.
Parameters that involve auxin production were multiplied by a factor 10^6 to avoid computer roundoff errors. Initial cell length is set to 10 μm.

Parameter	Initial values	Fitted values	Description	Script parameter name
λ	33	13	Growth factor ($\mu m \cdot h^{-1}$)	DATA.growthfact
A_0	1	2.56	Initial auxin concentration in cells ($\cdot 10^{-6} \cdot \mu g \cdot \mu m^{-1}$)	DATA.A0
μ_{col}	20	952	auxin production of Col0 ($\cdot 10^{-6} \cdot \mu g \cdot h^{-1}$)	DATA.wtmu
$\mu_{b1/b19}$	20	958	auxin production of abcb ($\cdot 10^{-6} \cdot \mu g \cdot h^{-1}$)	DATA.abcbmu
μ_{sav3}	5	554	auxin production of sav3 ($\cdot 10^{-6} \cdot \mu g \cdot h^{-1}$)	DATA.savmu
η	0.5	3.5	auxin degradation parameter ($\mu m \cdot h^{-1}$)	DATA.nu
P_{top}	0.5	0.46	Cell permeability on top side (h^{-1})	DATA.Dtop
P_{bottom}	1	1	Cell permeability on bottom side (h^{-1})	DATA.Dbottom
P_{in}	0.1	0.05	Cell permeability on inward side (h^{-1})	DATA.Din
P_{out}	0.1	0.13	Cell permeability on outward side (h^{-1})	DATA.Dout
ϵ	0.3	0.46	Remaining Pfr during night	DATA.remainFR
$P_{top(b1/b19)}$	0.5	0.17	Cell permeability on top side for b1/b19 mutant (h^{-1})	DATA.abcbDtop
$P_{bottom(b1/b19)}$	1	0.78	Cell permeability on bottom side for b1/b19 mutant (h^{-1})	DATA.abcbDbottom
$P_{in_{(b1/b19)}}$	0.1	0.12	Cell permeability on inward side for b1/b19 mutant (h^{-1})	DATA.abcbDin
$P_{out(b1/b19)}$	0.1	0.14	Cell permeability on outward side for b1/b19 mutant (h^{-1})	DATA.abcbDout
P_{cortex}	1	1.84	Cell permeability of each side of cortex cells (h^{-1})	DATA.Dcortex
$I_{fr,max}$	5	2320	Relative FR light intensity ($I_{fr,max} = 1$ in white light)	DATA.uFR
β	2	11	Natural Red/Far-Red ratio	DATA.nRFR
I_{c0}	150	31592	Initial cumulative light (μE)	DATA.Qlight0

a_1	$(1)^2$	$(1.21)^2$	S_1 sigmoid function parameter for growth sensitivity to auxin $((\mu g\cdot\mu m^{-1})^2)$	DATA.Asens
a_2	$(1)^2$	$(4.5)^2$	S_2 sigmoid function parameter for auxin production relative to R/FR ratio	DATA.ratioRFR
a_3	$(100)^2$	$(57)^2$	S_3 sigmoid function parameter for auxin degradation by light intensity $((\mu E)^2)$	DATA.Klight
u_μ	0.01	0.02	Translational parameter for function S_1 $(\mu g\cdot\mu m^{-1})$	DATA.mudecay
u_{fr}	0.01	5.99	Translational parameter for function S_2	DATA.decayRFR
u_I	0.01	23	Translational parameter for function S_3 (μE)	DATA.Mtdelay
b_1	10^7	110190	M_1 Monod function parameter for organ formation relative to light accumulation (μE)	DATA.Mtcumul
u_c	0.01	809	Translational parameter for function M_1(μE)	DATA.Mtdelay
